# Perceptions and attitudes of midwives on respectful maternity care during childbirth: a qualitative study in three district hospitals of Kigali City of Rwanda

**DOI:** 10.11604/pamj.2023.46.110.40764

**Published:** 2023-12-19

**Authors:** Valentine Uwamahoro, Jean Paul Sengoma Semasaka, Albert Ndagijimana, James Humuza

**Affiliations:** 1 University of Rwanda, College of Medicine and Health Sciences, School of Public Health, Kigali, Rwanda,; 2 Department of Clinical Sciences, Obstetrics and Gynecology, Umeå University, Faculty of Medicine, Umeå, Sweden

**Keywords:** Perceptions, attitudes, midwives, respectful maternity care, childbirth, Rwanda

## Abstract

**Introduction:**

Respectful Maternity Care (RMC) is “a universal human right for every childbearing woman”. In Rwanda, few studies conducted on RMC assessed how women perceive care provided during childbirth, yet little is known about providers' perspectives. We investigated the perceptions and attitudes of midwives towards the provision of RMC to complement women's viewpoints.

**Methods:**

this qualitative study used individual in-depth interviews in Kinyarwanda language. A purposive sampling method was used to reach out to twenty-eight midwives from three district hospitals in Kigali City. Transcribed interviews were translated into English and thematic content analysis was performed using Atlas Ti, version 7. The University of Rwanda College of Medicine and Health Sciences Institutional Review Board (Ref: 363/CHMS/IRB/2019) ethically approved this study before data collection.

**Results:**

the majority of participants revealed that they have knowledge on RMC and perceive that they provide maternal health care based on women´s rights. Positive attitudes towards providing RMC were reported by midwives, however, a considerable number of participants reported the existence of abusive practices. The majority of midwives reported facing many challenges affecting their ability to provide respectful maternal care.

**Conclusion:**

midwives understand the seven rights of women and have a positive attitude towards providing RMC. However, abusive practices still exist while providing RMC with considerable challenges, including overload and lack of labour monitoring materials. The adjustment of the ratio of midwives to clients and the availability of essential materials in labour monitoring is recommended to improve the quality of healthcare received by women during childbirth.

## Introduction

Pregnancy and childbirth are two periods that place women at substantial risk of mortality and morbidity, specifically in low- and middle-income countries (LMICs) [1]. Out of 303,000 maternal deaths that occurred worldwide in 2015, 99% were from LMICs [2]. The reduction in maternal and newborn mortality and morbidity is a global priority in sustainable development goals (SDGs) [3]. The number of health facilities with childbirth services as a measure to reduce maternal mortality remains insufficient [4,5]. Therefore, the consideration of physical health and overall well-being of pregnant women during childbirth becomes imperative to ensure a positive impact on maternal health outcomes [6]. Traumatic birth experience contributes to profound consequences on maternal well-being [7], including serious injuries and mistreatment both physically and emotionally with higher rates of maternal as well as neonatal and infant mortality and morbidity [8].

The concept of “respectful maternity care (RMC)” acknowledges that women's experiences of childbirth are vital components of health healthcare quality [8,9]. The World Health Organization (WHO) defines RMC as “care organized for and provided to all women in a manner that maintains their dignity, privacy, and confidentiality, while ensuring freedom from harm and mistreatment, and enables informed choice and continuous support during labour and childbirth” [8,10]. According to the stated universal rights of childbearing women charter, every woman has the right to dignified, respectful sexual and reproductive healthcare, including during childbirth [11]. However, operational components of RMC in terms of specific behaviours, practices, or standards in research and program implementation are often variable [12]. Mistreatment during childbirth can represent a violation of women's fundamental rights and serves as a powerful disincentive for women to seek healthcare in facilities for their subsequent deliveries and cannot be understood unless deep exploration is done on women's and health care providers' attitudes [1]. Therefore, helping women to have a positive childbirth experience is important for the woman's wellbeing and facilitates the mother-child bonding [13].

In September 2014, the WHO statement called for greater research, action, advocacy and dialogue on this important public health issue to ensure safe, timely, respectful care during childbirth for all women [11]. Disrespectful and abuse during childbirth may include physical abuse, non-consented clinical care, non-confidential care, non-dignified care, discrimination, abandonment, and detention in health facilities; and this cannot be understood unless deep exploration is done on women's and health care providers' attitudes [1]. There is a need to address this issue by providing RMC with positive impact on women´s labour and childbirth experience [14]. The only direct observational study conducted in of health facilities in East and Southern Africa, including Rwanda, to determine the prevalence of RMC found many women who had experienced poor interactions with providers and were not well-informed about their care, with both physical and verbal, with abandonment and neglect as the most frequent form of disrespect and abuse in the open-ended comments [9].

In Rwanda, a few studies conducted in regard to RMC during childbirth assessed the perceptions of women in the post-partum period on RMC offered by midwives through their childbirth experience [13,15]. Apart from the labouring women's perspective, to improve the quality of care during childbirth providers' respectful behaviour is another essential component to be considered [12]. However, little is known about healthcare professionals´ perceptions and attitudes towards RMC during childbirth in Rwanda. This study provided information on midwives´ perceptions and attitudes during childbirth, completed the information provided by women in previous studies and showed the area for improvement in maternal and child healthcare. This study aimed to understand the perceptions and attitudes of midwives towards the provision of RMC during childbirth. Specifically, it sought to investigate midwives' knowledge on the rights of women during childbirth, to identify midwives' attitudes towards respectful maternal care during childbirth, and to investigate midwives´ knowledge on various kinds of mistreatment experienced by women during childbirth period.

## Methods

**Study design:** this was a qualitative cross-sectional study, with an interview guide based on seven rights of women during childbirth, to gather the information about the perceptions and attitudes of midwives towards RMC during childbirth.

**Settings:** we conducted this study in three district hospitals from Kigali City: Masaka district hospital of Kicukiro district, Kacyiru district hospital of Gasabo district and Muhima district hospital of Nyarugenge district. The study area was selected due to a sizeable number of deliveries that take place in the City of Kigali, with many midwives; thus, expecting to gather various views among midwives who assist those deliveries. In a district where there are two district hospitals, we randomly selected one. We conducted data collection from1^st^ June to 15^th^ September 2019.

**Participants:** we conveniently included in the study all midwives who were working in labour or delivery ward at the time of the study, in each selected district hospital, and who were able to communicate in Kinyarwanda or English. We excluded midwives on sick or annual leave and those who were absent during the time of data collection.

### Data sources and measurement

We trained six data collectors, two per district (one moderator and one note taker). We used an interview guide including questions about the perceptions, attitudes, and practices of midwives during birth to collect data. Interviewers took field notes during interview to gather verbal and non-verbal information together with voice recording to capture all information. As a standard procedure, we reserved a secure place for interview based on their availability and preference to avoid coercion. Each participant meeting inclusion criteria was enrolled in the study. Data collection continued with the following available participant until we reached the theoretical saturation. At the end of our data collection, we found that our sample size was between eight to ten midwives. At the end of data collection, we conducted a debrief session on daily basis with all data collectors who met at a specific location and time to ensure the quality of data collected. We did this through discussions related to the interview guide, challenges of data, and review of notes taken during the interviews.

**Bias:** we selected participants based on one shared characteristic which is their professional recognition as registered midwives. Their role of providing maternal care to all attended women in labour, strengthens their ability of providing the richest information needed for our study. We trained data collectors on interview guide prior to data collection, they were not midwives and not staff in the selected hospitals or study investigators. Having participants knowledgeable on the subject and trained data collectors who master the tools was an added value to reduce bias from this study. The transcribes and final report were share with study respondents for validation.

**Study size:** this was a qualitative study thus we applied a theoretical saturation principle for data collection. During data collection, we interviewed all midwives falling under the study inclusion criteria and the interviewer had to stop collecting information when new data no longer bring additional insights to the research questions. At the end of data collection, we found that our sample size was between eight to ten midwives.

### Data analysis

We performed a verbatim transcription of interviews, followed by their expansion with field notes and translation of Kinyarwanda transcripts in English by the researchers. Data analysis followed the basic steps in qualitative data analysis. We read the transcripts and reread until the researcher got familiar with the content. The authors reviewed a few transcripts to identify and code concepts. We reviewed the transcripts by all co-authors separately and discussed in group how to refine codes based on study objectives and we created a codebook. We analysed the translated text through gathering information around the key themes, thematic analysis, highlight the differences and similarities within codes and categories using inductive and deductive reasoning. We coded themes and we identified them in a separate file. From the theme in a coding sort, the researcher interpreted and displayed data in a way that she detailed data from each code. From the details made from different themes in the created codes, the researcher was able to reduce data whereby only the essential concepts and interpretation were easy by explaining data´s core meaning. Finally, we identified a theme, based on the underlying meaning throughout the codes, with three categories and seven sub-categories. To seek agreement and validation on findings, the analysed document was been shared within research group until consensus was achieved. ATLAS Ti software was used in the process of data analysis whereby codes were matched to the content from provided responses, an output produced with codes and quotation was used in analysis.

To ensure confidentiality, we trained the research team on confidentiality and procedures to protect participant information. We did not share identified information otherwise a written permission from participants could be obtained unless it is among research team members and with password protected documents. Anonymity for the interviewees was our concern. We stored all identifying information in locked place. We used audio recordings for the purpose of the transcription step and we will destroy them upon publication of the study findings. We obtained a written informed consent from all participants in the study. We verbally explained the main objectives, procedures, risks, and benefits of the study for the interviewees. The researcher also allowed participants to read the information sheet for the purpose of allowing them asking questions for better understanding. We provided all participants with an informed consent form that also detailed all process of the study including audio recording. We did not consider the refusal of audio-recording as exclusion criteria to the study. For participants who did not consent for audio-recording, both the participated and the investigator took note instead.

Prior to conducting the interview, we fully educated the participants on the study, potential benefits to them and the community; voluntary participation and withdrawal, the use of audio recorder, data management and confidentiality. There was no risk to participate. Participants consented verbally and we obtained a written consent prior to starting the interview. We stored the written and recorded data in a locked place and soft data was protected using a password.

## Results

**Characteristics of study participants:** twenty-eight midwives were interviewed. All of them were aged 25-38 years (mean age 31.3 years) half of them were married and a half were single. The majority had worked for more than three years (n=14), ten had worked between one and three years and six had worked for less than one year. The great majority of participants were registered A1 Midwives (n=24) and four were A0 registered.

### Midwives' knowledge on respectful maternity care during childbirth

The RMC was launched in 2011 by White Ribbon Alliance and from this time it rooted in the international human right. The RMC community built a document, the human right of childbearing women (RMC Charter), with a common goal to demonstrate the respectful maternity care application as a fundamental human right in maternal health context. It has been used globally as a tool to help healthcare providers how maternity can be performed in accordance with the human right respect. It is in this purpose that it is used in this study to help researchers assessing perceptions and attitudes of midwives in providing RMC in selected settings.

### Midwives' awareness on respectful maternity care

In general, midwives working in maternity services from Masaka district hospital, Kacyiru district hospital and Muhima district hospital were knowledgeable on the RMC. *“This means that the woman should be cared of safely to ensure that she returns home alive with her child, she should receive standardized care based on her needs and cared with empathy without shouting on her.”* (Participant 7).

Midwives confirmed that they have had a course covering RMC during their curriculum, they have internal regulation and an orientation form guiding them about what information they need to share with their clients including their right to information, privacy, and confidentiality. *“Yeah, we have learnt those rights in ethical course, and we have internal regulations. We all know that clients need to be respected, informed about the procedure and confidentiality. For example, every client has her file; I am not allowed to share information from her file to others as it is her own information. Sharing it with others is prohibited.”* (Participant 18).

In addition, RMC has a few similarities with the existing clients´ rights available in each health setting. *“Women's rights are maybe those nine client´s rights including right to care, to information, to pain killer, to safe environment, eeh I may not recall all of them, but every healthcare provider must respect them.”* (Participant 2).

### Knowledge on the seven rights of Respectful Maternity Care

Most participants understood well seven rights which the rights are to be free from harm and ill treatment. *“In my understanding, respectful maternity care means providing care for every woman without any harm.”* (Participant 1).

There is right to information, informed consent and refusal, and respect for a woman´s choices and preferences, including companionship during maternity care. *“Let us say that, when the client enters the room, you need to provide explanation on each step of examination. For example, a pregnant woman comes in maternity for her first pregnancy; though she does not have any complication but she needs to be educated on how labour starts, the process of labour till delivery, the essential care for her child, the general management as well as some probable complications”*. (Participant 10).

There is right to privacy and confidentiality, the right to be treated with dignity and respect, the right to equality, freedom from discrimination, and equitable care, the right to healthcare and to the highest attainable level of health, and the right to liberty, autonomy, self-determination, and freedom from coercion; stated in RMC. *“When we talk about RMC, it means that every woman entering here receive care based on client´s right and the copy is available everywhere. Like other clients, she has right to be cared with dignity, respect, equitable manner and receive all healthcare needed as prescribed without any limit as well as considering her surrounding family.”* (Participant 8).

They make a self-presentation by introducing themselves to the clients and explain the basic information on the process of labour. Privacy was reported to be assured within existing infrastructure. *“I understand women´s rights as the right to be provided with explanations/information to her health problem, being treated as a human being, playing a role in her healthcare, privacy; so that she can feel satisfied with the service provided.”* (Participant 28).

In addition, participants perceived that they provide maternal healthcare services in equitable manner without any discrimination. For example, having health insurance or not does not affect the quality of service intended. *“From my experience, we provide healthcare for both insured and non-insured clients without counting on money as we need to save life and money comes later. This is the principal right but, in some situations, clients may not receive a full range of healthcare services based on payment issue, but I never observed it here, it may be in other hospital settings. We provide healthcare services and when client is unable to pay the social service intervenes.”* (Participant 2).

### Attitudes of midwives towards RMC during childbirth

Midwives have different point of view on their attitudes on RMC during childbirth. The majority report positive attitudes towards RMC and they provide respectful maternity care services by respecting its underlying rights. *“From the admission, the woman is informed about all process of labour including educating her about pain during contractions, we get her consent for any act proven by her signed informed consent, she is educated about feeding as many of them refuse to eat believing that it has bad impact during labour but we provide support to make sure that they are well fed. A midwife should always fix her/his eyes to the women to ensure close monitoring is done, check for contractions patterns, and it sometimes requires that you do not leave the woman alone depending on the stage.”* (Participants 4).

Participants agree that really midwives support women during childbirth by not blaming them when they cry during contractions, but they try to educate them on how they can cope with contractions. *“Yes of course.” We provide respectful maternity care. Let us take an example; I cannot examine a woman in presence of others due to the respect on her privacy. Indeed, I need to provide all needed services in general. Sometimes there are women who cry when they are feeling contractions; in this case you do not blame them, but we educate them on how she should cope with contractions.”* (Participant 23).

In another hand, a few participants reported having observed some negative attitudes among midwives while providing maternity healthcare services and the most reported is shouting on women. *“Though I am among young midwives, but I think I cannot say that I never seen such attitude. It happens and is not good, it is providing poor quality service. Yes, for example, we care about women with contractions; sometimes you give them advice or instructions, but they do not comply due to severe pain, therefore you talk to her in a bad manner or shout on her without considering her pain.”* (Participant 12).

Women are sometimes health cared in uncomfortable conditions, which is considered like unprofessional practice. *“Sometimes you can shout on the woman, or you do not provide the essential healthcare needed or performing suturing without anaesthesia and pain relief drugs and she may be in uncomfortable place. This also is not good for her.”* (Participant 11).

In addition to shouting on women and caring them in uncomfortable conditions, a midwives shared another negative attitude based on blaming women for varied reasons. *“This includes shouting on her during contractions and blaming her for not having medical insurance, not consulting medical care timely and not having information for what is happening from the point of entry.”* (Participant 20).

*“(...) like shouting on her for not complying to the advice, blaming her for not adhering to waiting room rules and you see that she is really disturbed by your instructions as some of the women may even manifest unusual behaviour or unexpected behaviour intentionally and they are blamed for that”*. (Participant 19).

### Midwives' knowledge of mistreatment experienced by women during childbirth

***Knowledge of kinds various of mistreatment experienced by women during the childbirth period:*** midwives had different views on existing kinds of women's mistreatment during childbirth period. They supported that women should be free from any harm during childbirth. The majority agreed that women need to be respected and they considered mistreatment as unprofessional practice.

*“In my understanding, this should sound bad like beating a pregnant woman. She is in pain and this pain is observed even from her appearance. So, I think we cannot cause harm to her, like shouting on her, even beating her. This is against the midwives´ practices.”* (Participant 2).

There are shared experiences in abusive practices among midwives which can be classified in three forms. The first reported form of abusive practice is verbal by shouting on women, but midwives reported that they do not do it deliberately. *“(....) people do not do it from their willing of shouting on women. Meeting with uncooperative woman sometimes creates a bad reaction because if you do not shout on her, the outcome may be bad and you are not able to save the life of the baby, but this is a rare situation.”* (Participant 25).

The second form of abusive practice is physical abuse by slapping woman particularly during second stage of labour and they considered it to stimulate and help them pushing for a positive labour outcome. *“This small room is not comfortable for women and midwives. The second stage is a difficult moment for both woman and midwives. You all wish to have a safe baby. You may decide to let the woman doing whatever she wants but you may regret later; you try your best to have a safe baby. It is better to be asked why you slapped a woman instead of responding to how the baby died. You do not slap a woman because you are against her, but for you need to have a safe baby.”* (Participant 26).

The third form is psychological, from missing labour support. A narrative report from one midwife provided information on lack of support with women in labour based on their health status like having a contagious disease. Midwives in this case do not provide labour support as usual for fearing being in contact with women by prohibiting holding them in arms. *“A woman in labour needs support and you need to react for every step positively. You need to be on her side but sometimes, considering a number of contagious diseases, a woman may feel contraction and want to be hold in arms or other support like back massage; in this case, some midwives shout on them.”* (Participant 16).

The provision of respectful maternity care across three district hospitals presents a few challenges: the medical system software and material, information, timely healthcare, privacy and infrastructure, client culture and religion, transfer process and others.

**Medical system software and material:** some of the users of community-based health insurance (CBHI) report that the process of check-up and registration into the system before receiving health care may cause a delay in intervention for some cases which need emergency and affects midwives who may be requested to advocate for women and go to explain the emergency case to the CBHI agent to provide the needed healthcare. *“This time, the system process requires for each insurer to be checked into system before receiving service as it is in conjunction with client identity card information. Here we always have a problem of internet; so that time, they cannot provide material or medicine to the client which delays the process as you are obliged to go yourself advocating for him/her while you have other responsibilities, and this is time consuming.”* (Participant 22).

In addition, the lack of labour monitoring material like cardiotocography CTG was reported by most of the participants and this result in late detection of foetal distress leads to increase in neonatal death rate. *“The main challenge in monitoring is the lack of cardiotocography (CTG). In our district hospital we do not have any. So, the consequence you come and check on the foetal heartbeat and you find baby dead inside from a woman who has been admitted with a live baby. This is what we call Intra-uterine foetal demise (IUFD).”* (Participant 21).

**Right to information:** some participants think that clients receive information correctly. However, most participants reported the shortage of midwives and high number of clients as a fact to lack of providing all needed information to women. *“The first challenge is that clients are too many considering to the number of midwives, beds, and rooms. It means, when you have many clients, you provide short information, you do not enter in detailed, and you cannot get another time to come back with a high number of activities. At this time, the client cannot get the chance for asking questions.”* (Participant 28).

**Timely healthcare:** most participants reported that, due to overloaded work, they do not receive timely healthcare. The most activity affected is regular foetal monitoring. *“I cannot say that clients receive healthcare timely at 100%. Depending on the high number of clients admitted. For example, hearing foetal heart rate among the women in room every 30minutes cannot be realized because you make a cycle, and you reach on the last too late”*. (Participant 6)

An existing strategy used for triage with distinct colour based on the severity of woman's condition is used whereby red symbolize an emergency case, the yellow colour symbolize the urgent case and green colour symbolize no-severe case, but this can lead to a delay in health healthcare provision. *“Depending on this high number, the clients should come at hospital early morning and receive healthcare late. The one scored in green or yellow, you do not start with her as she is not considered as an emergency case, but progressively her issue becomes severe and falls in red colour while if she has been admitted timely her condition should not be severe as it is.”* (Participant 6).

**Privacy and infrastructure:** participants report that they are aware of the importance of privacy and try their best to respect privacy with existing infrastructure. However, privacy was reported to be affected by short space in maternity ward and gynaecological beds. The pregnant women are closed to each other and are obliged to share rooms and beds. This interferes with midwives´ ability to provide confidential information to the women. *“There are many challenges. Let us talk about infrastructure and hospital capacity. When we receive many clients two women share the same bed which interferes with the privacy of the client, at that time the only thing to do as healthcare provider is to maximize the benefit from each one.”* (Participant 18).

**Transfer process:** an overview on some cases that are more likely to be transferred from District hospital to Referral Hospital reported include peritonitis, post-partum haemorrhage, severe pre-term labour, having more than two previous uterine scar and severe pre-eclampsia. There are requirements for transfer such as caution fee for those without the health insurance and availability of ambulance. Most participants report not having any challenges during external transfer process but there are few of them that revealed challenge like the lack of place in maternity ward which leads to refusal of transferred case with fact that there is no available place.

*“The first, do you think that transfer the client is easy? Our challenge for transfer to referral hospital is another issue. There are cases which are refused, and others are rejected from the criteria of admission like caution fee more likely to people without insurance which create a long discussion with delay.”* (Participant 19).

The information gathered from our participants suggests that the system of maternity health care service delivery is good, due to presence of key material and infrastructure; however, workload was persistently mentioned as major challenge.

## Discussion

This study aimed to investigate the perceptions and attitudes of midwives toward the provision of respectful maternity care during childbirth in three district hospitals in Kigali City. The main findings from this study reveal that midwives have knowledge of respectful maternity care from learned courses, internal regulations, and international standards. However, a few midwives reported existing abusive practices such as shouting and slapping women. This is similar to the findings from a qualitative systematic review and meta-synthesis done in sub-Saharan Africa whereby over half of midwives reported having no choice but to yell, slap, or neglect women to motivate them to push, using language like ‘need to’ or ‘forced to’ when describing their actions [16]. Most midwives assured their willingness to provide RMC in their workplace but they reported having challenges that were focused on their high workload with its impact on information sharing between midwives and women. This has some similarities with what was expressed by midwives in a qualitative study conducted in Iran on midwives' perspectives of respectful maternity care during childbirth which showed that midwives expressed their awareness of the importance of communicating information to women although this could be a challenge due to time pressure [12]. Midwives are knowledgeable about the seven rights during childbirth, and they admit the women after explaining to them about their rights on information. A qualitative study conducted in Ethiopia on promoting compassionate and respectful maternity care during facility-based delivery expressed similar perspective whereby the majority of the midwives sought to build and establish positive relationships with the women by engaging in conversational interactions and providing emotional support; and communication between the client and the midwife was reported as part of the compassionate and respectful midwifery care [10].

In this study, midwives shared their points of view on privacy, liberty, and freedom as stated in the internal regulation. They have an orientation form guiding them whereby the woman is required to sign after being explained the process of healthcare. A previous qualitative study conducted on exploring midwives´ understanding of respectful and non-abusive maternal care in Ghana demonstrated similar information on how midwives provide information to women which include their right to healthcare, to information, to privacy, to consent, to best healthcare, to choose to treatment and the possible consequence to the chosen treatment [10]. Understanding respectful maternity care implies considering every right among the seven rights stated in the RMC charter. All participants have knowledge on right to freedom from harmful practices and they are committed to providing maternal healthcare free from harm as a mean of creating a trustful environment between them and women. The same qualitative systematic review and meta-synthesis in sub-Saharan Africa found that midwives are proud to be called midwives and are so-called mamas because they have seen that providing respectful maternity care brings happiness for both midwives and women and they feel more enthusiastic which encourages women to come to the facility [16]. The right to information was reported to be respected while providing RMC by most of the participants but a few of them reported that there is a challenge in providing full information to our clients due to overloaded work. Similarly, a study conducted in Rwanda revealed that women with complications during labour were not informed about their conditions before discharge and this is supported by information from one participant who stated that she did not know much about her sickness. She was just confused and thought that she was going to die from her condition [17]. Another participant from another qualitative phenomenological study in Rwanda on the meaning of a poor childbirth experience done in Rwanda stated that the lack of explanations increased the feeling of being disrespectfully treated. She further said she felt like a cow taken to a slaughter house without any explanation as midwives kept looking at her silently which ignited fear in her and thoughts that made her wondered if she was going to die or that they were probably hiding important information from her [15].

The responses from our participants revealed that most maternal healthcare services provided to women are based on privacy rights but also few participants revealed that privacy is affected in many ways due to some challenges including sharing one bed and having women in labour in a general room. This is similar to a study conducted in Tanzania on the prevalence of disrespect and abuse during facility-based childbirth, which revealed that lack of privacy during childbirth stands at 53% [6]. A qualitative evidence synthesis on factors that influence the provision of intra-partum and postnatal healthcare by skilled birth attendants in low- and middle-income countries revealed some challenges in providing maternal healthcare respected privacy of the clients as stated by skilled birth attendants that when they have heavy workloads, and sometimes with difficult for them to provide privacy healthcare with all women [18]. The right to dignity and respect is reported to be respected by all participants but with a few challenges like overloaded work. This has been reported even in the qualitative evidence synthesis on factors that influence the provision of intra-partum and postnatal healthcare by skilled birth attendants in low- and middle-income countries whereby heavy workloads report the limited time that health workers had for conducting thorough assessments of women, and only women suspected to have complications were examined [18]. In our findings, few participants revealed that it happens that a woman should be neglected by midwives who may not be responding to women´s calls for help while discussing their own issues. This is supported by another study conducted in Rwanda on the meaning of poor childbirth experience which revealed that some women are neglected by health healthcare providers by refusing to be beside them during critical moments as explained by one participant that she was alone during labour, it happened for her to scream out to the nurse behind the curtain and the nurse didn´t come for help. When she tried to call again to explain that she was feeling the baby coming out, the nurse replied that she should continue pushing till she gave birth alone [15]. In addition to the work overload, our findings reveal that lack of materials like CTG machines for foetal monitoring and sometimes stock out on some medication affect the way midwives can provide timely healthcare. CTG machines are not available in all health care facilities in Rwanda, a limited resource country; but efforts are made to equip all service providers with quality materials to ensure the high quality of delivery services.

During discharge process, all midwives report that women are not retained at the hospital for payment issues because they all have social services that support women who cannot afford hospital bills. This is similar to the findings from a qualitative study exploring midwives´ understanding of respectful and non-abusive maternal healthcare in Ghana whereby midwives reported they have the social welfare in charge of discharged cases without payment as stated by the hospital policy [19]. But on another hand, we found that there are challenges for those women who are transferred to the highest level (tertiary level); it happens that they may be refused by the referral level depending on bed availability or health healthcare providers miscommunication. Despite the reported knowledge on how respectful healthcare is provided, there are existing abusive practices revealed by some participants. These practices are classified into physical and psychological abusive practices. For physical abusive practices, the most reported is slapping women during pushing time and few revealed episiotomy suture without anaesthesia. This is also reported in East and Southern Africa from a direct observation of respectful maternity care study in five countries which revealed that women are slapped during labour specifically during the second stage of labour. An example is seen when a midwife slapped a woman when it was difficult to deliver the placenta [9]. This kind of abusive practice is also supported by the findings from a study conducted in Ethiopia on service providers´ experiences of disrespectful and abusive behaviour towards women during facility-based childbirth which revealed that a quarter of the respondents had ever witnessed the use of physical force or abusive behaviour such as slapping or hitting during labour [10].

In our findings, midwives report that hitting women helps them to save their infants. This is supported by findings from Ghana revealing that women are hit for the purpose of life-saving, a bad practice that is not planned. Midwives reported that when the baby´s head is crowned, they must hit the childbearing woman because women tend to close their legs and midwives hit on them for the purpose of saving the baby´s life. They report that doing this is not wrong for them as it was intended to save, not harm [19]. For psychological form of abuse, in our findings, the most reported is shouting at women. Another study conducted in Rwanda on the meaning of poor childbirth experiences revealed that midwives shout at women especially during pushing time, which ends in doubting on the expected outcome [12,15].

Even though there are existing abusive practices and negative attitudes towards respectful maternal care, most of the midwives' report that respectful maternity care is not respected 100% but they try their best to reach the positive outcome of course by overcoming existing provided workplace challenges, through training of healthcare providers on respectful maternity care and advocate for increase of midwives´/women ratio and they estimated the quality-of-care RMC at 80-85%. This suggestion is also stated in the study conducted in Ethiopia “It is essential to address structural problems such as provider workload, and all other initiatives aimed at improving midwives´ interpersonal relationships with women to provide compassionate and respectful client-centred maternity care. Cooperation and synchronisation between researchers, health organisations and the Ethiopian government are also required to provide preservice and in-service training, improve working conditions, and streamline various processes to promote respectful care of women during childbirth” [10]. These findings also are comparable with the findings from a cross-sectional study conducted in Rwanda on the association between perceptions of healthcare and women's childbirth experience where 77.5% of the participants reported a good experience in maternal healthcare which increases the rate of access to maternal healthcare [13], and findings from a study on exploring provider perspectives on respectful maternity care in Kenya which stated that an improved provider management, communication, and teamwork and good communication between health care providers and clients has improved and goes beyond by reaching even on clients side which reduced some of their clients´ perceptions on disrespect and abuse [20].

**Study limitations:** this study was conducted in three district hospitals located in Kigali City based on their easy accessibility; therefore, the findings from this study cannot be generalized given that it did not include midwives from different level of health facilities.

## Conclusion

This qualitative study describes the perceptions and attitudes of midwives towards the provision of respectful maternity care during childbirth in three district hospital, Kigali City. The findings show that midwives understand well the seven rights of women as stated in the respectful maternal care charter. There are positive attitudes towards providing respectful maternal care such as providing information, privacy respect and providing all needed services, however there are also negative attitudes like shouting on women and treating them in uncomfortable condition that were reported by participants. Participants reported challenges associated with the provision of respectful maternity care which included high workload and lack of essential monitoring material like CTG. Therefore, there is a need to overcome the existing challenges in providing respectful maternity care by adjusting the midwives´/client´s ratio and providing essential material for labour monitoring to improve the quality of healthcare received by women during childbirth.

### What is known about this topic

- *Health facilities providing childbirth services as a proximate of ensuring sound maternal and child health are limited; this is believed to have contributed to unbearable childbirth experiences among mothers who felt violated and ill-treated within health facilities in LMICs;*
- *The prevalence of respectful maternity care has been determined among mothers in most Southern and Eastern African countries like Rwanda; current knowledge states the most frequent forms of disrespect and abuse include the performance of clinical procedures without women's consent and abandonment by midwives during labor;*
- *Women's perceptions of respectful maternity care in Rwanda are known; insights from women suggest quality improvement in terms of midwives' behavior towards women in labor*.

### What this study adds

- *We provide additional information on existing literature demonstrating that midwives in Rwanda have knowledge of the seven rights of women regarding maternal and child health as stated in the respectful maternal care charter, most of this knowledge on RMC according to them is learned at school;*
- *Majority of maternity service providers (midwives) in Rwanda have a positive attitude towards providing respectful maternal care as they are willing to provide information, respect women's privacy, and provide needed services;*
- *Midwives are knowledgeable about existing negative attitudes related to providing respectful maternity care such as shouting at women and treating them in an undesirable manner, including abusive practices like beating during labour; however, they reported factors leading to such negative attitudes include high workload and lack of essential monitoring material like CTG machine*.
